# A new method for the extraction of fetal ECG from the dependent abdominal signals using blind source separation and adaptive noise cancellation techniques

**DOI:** 10.1186/s12976-015-0021-2

**Published:** 2015-11-14

**Authors:** Abdelghani Ghazdali, Abdelilah Hakim, Amine Laghrib, Nezha Mamouni, Said Raghay

**Affiliations:** Department of Mathematics, Laboratory of Applied Mathematics and Computer Science (LAMAI), FSTG, Cadi Ayaad University, Abdelkarim Elkhattabi, Marrakech, 40000 Morrocco

**Keywords:** Blind source separation, Fetal electrocardiogram extraction, Copula, Bilateral TV filter, Dependent sources

## Abstract

**Background:**

The electrocardiogram (ECG) is a diagnostic tool that records the electrical activity of the heart, and depicts it as a series of graph-like tracings, or waves. Being able to interpret these details allows diagnosis of a wide range of heart problems. Fetal electrocardiogram (FECG) extraction has an important impact in medical diagnostics during the mother pregnancy period. Since the observed FECG signals are often mixed with the maternal ECG (MECG) and the noise induced by the movement of electrodes or by mother motion, the separation process of the ECG signal sources from the observed data becomes quite complicated. One of its complexity is when the ECG sources are dependent, thus, in this paper we introduce a new approach of blind source separation (BSS) in the noisy context for both independent and dependent ECG signal source. This approach consist in denoising the observed ECG signals using a bilateral total variation (BTV) filter; then minimizing the Kullbak-Leibler divergence between copula densities to separate the FECG signal from the MECG one.

**Results:**

We present simulation results illustrating the performance of our proposed method. We will consider many examples of independent/dependent source component signals. The results will be compared with those of the classical method called independent component analysis (ICA) under the same conditions. The accuracy of source estimation is evaluated through a criterion, called again the signal-to-noise-ratio (SNR). The first experiment shows that our proposed method gives accurate estimation of sources in the standard case of independent components, with performance around 27 dB in term of SNR. In the second experiment, we show the capability of the proposed algorithm to successfully separate two noisy mixtures of dependent source components - with classical criterion devoted to the independent case - fails, and that our method is able to deal with the dependent case with good performance.

**Conclusions:**

In this work, we focus specifically on the separation of the ECG signal sources taken from skin two electrodes located on a pregnant woman’s body. The ECG separation is interpreted as a noisy linear BSS problem with instantaneous mixtures. Firstly, a denoising step is required to reduce the noise due to motion artifacts using a BTV filter as a very effective one-pass filter for denoising. Then, we use the Kullbak-Leibler divergence between copula densities to separate the fetal heart rate from the mother one, for both independent and dependent cases.

## Background

During pregnancy, monitoring the mother and fetus heart condition is primordial to collect information about their health and diagnose possible diseases. A very popular way to obtain the fetal ECG is recording it through skin electrodes attached to the mother’s abdomen [1, 2]. The process of recording is usually complicated since the MECG is with higher amplitude compared with the fetal one. In addition, the FECG is contaminated by many sources of noise such as the electronic equipment, the patient respiration and movement [3]. However, an effective signal processing study is needed in order to separate magnificently the two wanted sources FECG and MECG components from the corrupted mixture recordings. Since 1960, many signal-processing techniques have been introduced to improve the quality of the FECG detection with varying average of success [4–6]. The most popular techniques include adaptive filters [3], singular-value decomposition (SVD) [7], wavelet transform [8], adaptive Neuro-Fuzzy inference systems to treat the nonlinear relationship between the thoracic ECG and the maternal ECG component in the abdominal ECG signals [5]. Another efficient work was the use of blind source separation (BSS) [9]. The BSS aims to recover unknown source signals from a set of observations which are unknown mixtures of source signals. In order to separate the mixtures, different assumptions on the sources have to be made. In the literature, the most common assumptions are statistical independence of the source components and the condition that at most one of the components is gaussian. Under these assumptions, the BSS problem is linked to the well known problem of Independent Component Analysis (ICA), see for instance [10]. Further, it has been shown in [11] that, based on copula models, without the assumption of the independence of the source components, we can still identify both mixing matrix and sources uniquely (up to scale and permutation indeterminacies) of (free-noisy) mixtures of both independent and dependent source components. Motivated by various cases where the ECG signals are dependent, we investigate, in the present paper, models of noisy linear instantaneous mixtures of independent/dependent sources, for which we propose, based on the previews works [11], a new BSS procedure. Indeed, the dependence may be a consequence of numerous factors, for example, when the heartbeat of the mother and foetus coincides.

The prposed technique is divided in two stages : the first one is a denoising process using a bilateral TV [12, 13], while the second stage is about to separate the fetal heart rate from the mother one, using the modified Kullbak-Leibler divergence between copula densities. The paper is organized as follows: In “Methods” Section “Principle of BSS”, we present the general formulation of the blind source separation in a noisy context, and in Section “Proposed approach” of “Methods”, we describe the two steps of this proposed method. In “Results and discussion”, we present the performance of the proposed algorithm on both dependent and independent ECG signals. Finally, we end this paper by a conclusion.

## Methods

## Principle of BSS

In this paper, we consider the noisy linear BSS model with instantaneous mixtures, the operator ***A*** corresponds then to a scalar matrix, and we assume that the number of sources is equal to the number of observations. The model writes

(1) $$  \bar{\boldsymbol{x}}(t):=\boldsymbol{A}\,\boldsymbol{s}(t)+ \boldsymbol{n}(t), \;t \in \mathbb{R},  $$

where $\boldsymbol {\bar {x}} \in \mathbb {R}^{p}$ is the vector of observations, $\boldsymbol {s} \in \mathbb {R}^{p}$ is the unknown vector of sources to be estimated, $\boldsymbol {n} \in \mathbb {R}^{p}$ is the vector noise, and ***A*** is the unknown mixing matrix. The Eq. (1) can also be written as

(2) $$  \boldsymbol{\bar{x}}(t):=\boldsymbol{x}(t)+\boldsymbol{n}(t),  $$

with ***x***(*t*)=***As***(*t*) is the noise-free mixed vector signals. The aim here, is to estimate the sources ***s***(*t*) using only the observations $\boldsymbol {\bar {x}}(t)$. The sources are recovered using the following linear separating system

(3) $$  \boldsymbol{\bar{y}}(t):=\boldsymbol{n}\,\boldsymbol{\bar{x}}(t), \; t \in \mathbb{R},  $$

where $\boldsymbol {\bar {y}}(t)\in \mathbb {R}^{p}$ is the noisy estimate of ***s***(*t*), and $\boldsymbol {n}\in \mathbb {R}^{p \times p}$ is the separating matrix. In noisy BSS, we come across the problem of the estimation of the noise-free sources components; following Eq. (3), we only get noisy estimate of source. Therefore, we would like to obtain estimates of the original sources ***s***(*t*) with minimal noise. In other words, it is not enough to estimate the mixing matrix, through (3). The estimated source signals obtained by a direct BSS, for the noisy case, can be written as follows

(4) $$  \begin{aligned} \boldsymbol{\bar{y}}(t)&:=\boldsymbol{n}\,\boldsymbol{\bar{x}}(t)\\ &:= \boldsymbol{y}(t) + \boldsymbol{\bar{n}}(t), \end{aligned}  $$

where ***y***(*t*):=***BAs***(*t*) and $\boldsymbol {\bar {n}}(t)=\boldsymbol {B}\,\boldsymbol {n}(t)$. That is the noisy estimated source $\boldsymbol {\bar {y}}(t)$ is the sum of ***y***(*t*) the ideal estimated source, and the noise $\boldsymbol {\bar {n}}(t)$. Ideally, we would like to retrieve ***y***(*t*) by denoising $\boldsymbol {\bar {y}}(t)$, but it’s rather difficult since the noise $\boldsymbol {\bar {n}}(t)$ is unknown.

During last years, several algorithms have been proposed to tackle the noisy BSS problem. In [14], the authors propose a two-step approach by combining the fraction allower order statistic for the mixing estimation and minimum entropy criterion for noise-free source component estimation. In [15], a whitening procedure is proposed to reduce the noise effect. The proposed method is based on two steps: (*i*) denoising of the observed signal $\boldsymbol {\bar {x}}(t)$ before demixing; (*ii*) a simultaneous BSS procedure via minimization of the modified Kullbak-Leibler divergence between copula densities.

## Proposed approach

The proposed approach proceeds in two steps: Step 1: uses the bilateral TV model for denoising the observed signals. Step 2: uses copula as the basic BSS block, which finds the separating matrix, thus estimating the source signals.

### Step 1: signal denoising

We consider in this step the denoising problem using the bilateral filter [16]. Let $\boldsymbol {\bar {x}}(t)\in \mathbb {R}^{p}$ be the noisy observed random vector signal, which is related to the ideal observed one: $\boldsymbol {x}(t)\in \mathbb {R}^{p}$, using the following formulation

$$\boldsymbol{\bar{x}}(t):=\boldsymbol{x}(t)+\boldsymbol{n}(t). $$

Via Bayes rule, finding the ideal signal ***x*** is equivalent to solve the minimization problem (5) using the Maximum a posteriori (MAP) [13].

(5) $$\begin{array}{@{}rcl@{}} \boldsymbol{x} &=& \underset{\boldsymbol{x}}{\text{argmax}} \left\{p(\boldsymbol{x}/\boldsymbol{\bar{x}}) \right\} \\ &=& \underset{\boldsymbol{x}}{\text{argmax}} \left\{\frac{p(\boldsymbol{\bar{x}}/\boldsymbol{x}).p(\boldsymbol{x})}{p(\boldsymbol{\bar{x}})}\right\} \\ &=&\underset{\boldsymbol{x}}{argmin}\left\{-log (p(\boldsymbol{\bar{x}} /\boldsymbol{x}))- log(p(\boldsymbol{x}))\right\} \end{array} $$

where $p(\boldsymbol {\bar {x}} /\boldsymbol {x})$ represents the likelihood term and *p*(***x***) denotes the prior knowledge in the ideal signal ***x***. To solve this problem we need to describe the prior Gibbs function (p.d.f) *p*. In this work, we use the bilateral filter TV as a p.d.f function since it is computationally cheap to implement, and preserves the signal information. The expression of bilateral p.d.f looks like

(6) $$ p(\boldsymbol{x})=\exp \left(-\lambda \sum\limits_{j=-m}^{m} \alpha^{|j|} \| \boldsymbol{x}-\boldsymbol{G}^{j}\boldsymbol{x} \|_{1}\right),   $$

where ***G***^*j*^ implies a shift right of *j* samples. The scalar weight *α* (0<*α*<1), is applied to give a spatially decaying effect to the summation of the regularization terms. *m* is the spatial window size and *λ* the regularisation parameters.

Using the Eq. (6) and by substituting the expression of *p*(.) in the Eq. (5), the solution for denoising the vector ***x*** is then given through the minimization problem

(7) $$  \underset{\boldsymbol{x}}{\inf}\, \left\{\frac{1}{2}\int_{\mathbb{R}^{p}}|\boldsymbol{x}(t)-\boldsymbol{\bar{x}}(t)|^{2}\mathrm{d}t + \lambda \sum\limits_{j=-m}^{m} \alpha^{|j|} \| \boldsymbol{x}-\boldsymbol{G}^{j}\boldsymbol{x} \|_{1} \right\},\qquad \lambda >0.  $$

The first term in (7) measures the fidelity to the data, the second is a smoothing term that controls the variation of ***x***. The problem (7) admits a unique solution in the space of bounded variation (BV) [16]. Computationally, the model (7) is usually solved by its formal Euler-Lagrange equation but the convergence it hardly assured. To avoid this illness, the minimizer of the corresponding discrete problem will be presented and solved using the Primal-Dual algorithm [17], in Section “The denoising of the discrete observed signal” hereafter.

### Step 2: Separation of the MECG and FECG signals

The aim of the following step is to reconstruct an estimated source signal ***y***(*t*) from the denoised observed signal ***x***(*t*). It has been shown in [11] that if we dispose of some prior information about the density copula of the random source vector ***s***(*t*), we can detect both the mixing matrix and the sources uniquely for both independent and dependent sources. Let $\boldsymbol {Y} := (Y_{1},\ldots,Y_{p})^{\top } \, \in \mathbb {R}^{p},\,p\geq 1$, a random vector, with cumulative distribution function (c.d.f.)

(8) $$  F_{\boldsymbol{Y}}(\cdot):\,\boldsymbol{y}\in \mathbb{R}^{p} \mapsto F_{\boldsymbol{Y}}(\boldsymbol{y}):=F_{\boldsymbol{Y}}(y_{1},\ldots, y_{p}):=\mathbb{P}\left(Y_{1}\leq y_{1},\ldots,Y_{p}\leq y_{p}\right),  $$

and continuous marginal functions

(9) $$  F_{Y_{i}}(\cdot):\,y_{i}\in \mathbb{R} \mapsto F_{Y_{i}}(y_{i}):=\mathbb{P}(Y_{i}\leq y_{i}), \, \forall i=1,\ldots, p.  $$

The mutual information of ***Y*** is defined by

(10) $$  MI(\boldsymbol{Y}):=\int_{\mathbb{R}^{p}} -\log \frac{\underset{i=1}{\overset{p}{\prod}}f_{Y_{i}}(y_{i})}{f_{\boldsymbol{Y}}(\boldsymbol{y})} f_{\boldsymbol{Y}}(\boldsymbol{y}) \, \mathrm{d}y_{1},\ldots, \mathrm{d}y_{p}.  $$

It is called also the modified Kullbak-Leibler divergence (*KL*_*m*_), between the product of the marginal densities and the joint density of the vector. Note also that $MI(\boldsymbol {Y}):= KL_{m} \left (\underset {i=1}{\overset {n}{\prod }}f_{Y_{i}},f_{\boldsymbol {Y}} \right)$ is nonnegative and achieves its minimum value zero iff $f_{\boldsymbol {y}}(.)=\underset {i=1}{\overset {p}{\prod }}f_{Y_{i}}(.)$ i.e., iff the components of the vector ***Y*** are statistically independent. To clarify more precisely the BSS step, we will study separately, the case where the source components are independent, and the case where the source components are dependent.

#### Independent source components

Recall that the relationship between the probability density function and copula density is given by

(11) $$  f_{\boldsymbol{Y}}(\boldsymbol{y})=\underset{i=1}{\overset{p}{\prod}}\,f_{Y_{i}}(y_{i})\boldsymbol{c}_{\boldsymbol{Y}}\left(F_{Y_{1}}(y_{1}),\ldots,F_{Y_{p}}(y_{p})\right).  $$

Assume that the source components are independent. Using the relation (11), between and applying the change variable formula for multiple integrals, we can show that *MI*(***Y***) can be written via copula densities as

(12) $$  MI(\boldsymbol{Y}):=\int_{[0,1]^{p}} -\log\left(\frac{1}{\boldsymbol{c}_{\boldsymbol{Y}}(\boldsymbol{u})} \right)\boldsymbol{c}_{\boldsymbol{Y}}(\boldsymbol{u})\,\mathrm{d}u=:KL_{m} \left(\boldsymbol{c}_{\scriptscriptstyle{\prod}},\boldsymbol{c}_{\boldsymbol{Y}}\right),  $$

where ***c***_***Y***_(***u***) is the density copula of ***Y***, and $\boldsymbol {c}_{\scriptscriptstyle {\prod }}(\boldsymbol {u}):=1_{[0,1]^{p}}(\boldsymbol {u})$ is the product copula density. Moreover, $KL_{m} \left (\boldsymbol {c}_{\scriptscriptstyle {\prod }},\boldsymbol {c}_{\boldsymbol {Y}}\right)$ is nonnegative and achieves its minimum value zero iff $\boldsymbol {c}_{\boldsymbol {Y}}(\boldsymbol {u})=\boldsymbol {c}_{\scriptscriptstyle {\prod }}(\boldsymbol {u}),\, \forall \boldsymbol {u} \in [0,1]^{p}$, namely, iff the components of the vector ***Y*** are independent.

Our approach consists in minimizing with respect to ***B***, the following separation criterion:

(13) $$  KL_{m} \left(\boldsymbol{c}_{\scriptscriptstyle{\prod}},\boldsymbol{c}_{\boldsymbol{Y}}\right):=\mathbb{E} \left[\log\left(\frac{c_{\boldsymbol{Y}}\left(F_{Y_{1}}(Y_{1}),\ldots, F_{Y_{p}}(Y_{p}) \right)}{\boldsymbol{c}_{\scriptscriptstyle{\prod}}\left(F_{Y_{1}}(Y_{1}),\ldots, F_{Y_{p}}(Y_{p})\right)} \right)\right],  $$

where $\mathbb {E}(\cdot)$ denotes the mathematical expectation. The function $\boldsymbol {B} \mapsto KL_{m} \left (\boldsymbol {c}_{\scriptscriptstyle {\prod }},\boldsymbol {c}_{\boldsymbol {Y}}\right)$ is nonnegative and attains its minimum value zero at ***B***=***DPA***^−1^, where ***D*** and ***P*** are, respectively a diagonal and permutation matrix. In other words, the separation is achieved in $\boldsymbol {B} = \underset {\boldsymbol {B}}{\arg \min }\, KL_{m} \left (\boldsymbol {c}_{\scriptscriptstyle {\prod }},\boldsymbol {c}_{\boldsymbol {Y}}\right)$.

#### Dependent source components

In the case where the source components are dependent, we assume that we dispose of some prior information about the density copula of the random source vector ***s***. Note that this is possible for many practical problems, it can be done, from realizations of ***s***, by a model selection procedure in semiparametric copula density models $\left \{ \boldsymbol {c}_{\boldsymbol {\theta }}(\cdot);\, \boldsymbol {\theta } \in \boldsymbol {\theta }\subset \mathbb {R}^{d}\right \}$, typically indexed by a multivariate parameter ***θ***, see [18]. The parameter ***θ*** can be estimated using maximum semiparametric likelihood, see [19]. We denote by $\widehat {\boldsymbol {\theta }}$, the obtained value of ***θ*** and $\boldsymbol {c}_{\widehat {\boldsymbol {\theta }}}(\cdot)$ the copula density modeling the dependency structure of the source components. Obviously, since the source components are assumed to be dependent, $\boldsymbol {c}_{\widehat {\boldsymbol {\theta }}}(\cdot)$ is different from the density copula of independence $\boldsymbol {c}_{\scriptscriptstyle {\prod }}(\cdot)$. Hence, we naturally replace in (12), $\boldsymbol {c}_{\scriptscriptstyle {\prod }}$ by $\boldsymbol {c}_{\widehat {\boldsymbol {\theta }}}$, then we define the separating criterion

(14) $$  \begin{array}{ll} KL_{m} \left(\boldsymbol{c}_{\widehat{\boldsymbol{\theta}}},\boldsymbol{c}_{\boldsymbol{Y}}\right)&:=\int_{[0,1]^{p}} -\log\left(\frac{\boldsymbol{c}_{\widehat{\boldsymbol{\theta}}}(\boldsymbol{u})}{\boldsymbol{c}_{\boldsymbol{Y}}(\boldsymbol{u})} \right)\boldsymbol{c}_{\boldsymbol{Y}}(\boldsymbol{u})\,\mathrm{d}\boldsymbol{u}\\ &:=\mathbb{E} \left[ \log\left(\frac{\boldsymbol{c}_{\boldsymbol{y}}(F_{Y_{1}}(Y_{1}),\ldots, F_{Y_{p}}(Y_{p}))}{\boldsymbol{c}_{\widehat{\boldsymbol{\theta}}}(F_{Y_{1}}(Y_{1}),\ldots, F_{Y_{p}}(Y_{p}))} \right)\right]. \end{array}  $$

Moreover, we can show that the function $\boldsymbol {B} \mapsto KL_{m} \left (\boldsymbol {c}_{\widehat {\boldsymbol {\theta }}},\boldsymbol {c}_{\boldsymbol {Y}}\right)$, is nonnegative and attains its minimum value zero at ***B***=***DPA***^−1^. The separation for dependent source components, is reached in $\boldsymbol {B} = \underset {\boldsymbol {B}}{\arg \min }\, KL_{m} \left (\boldsymbol {c}_{\widehat {\boldsymbol {\theta }}},\boldsymbol {c}_{\boldsymbol {Y}}\right)$.

## Statistical estimation

### The denoising of the discrete observed signal

In this section, we show how to estimate, in practice, ***x*** from the noisy observation $\boldsymbol {\bar {x}}$. Recall that this estimation is obtained by solving the discrete version of the problem (7) using Primal-Dual algorithm. We start with the following notation :

(15) $$  K=\lambda \sum\limits_{j=-m}^{m} \alpha^{|j|} (I-\boldsymbol{G}^{j}),  $$

and

(16) $$  \mathcal{F}(K\boldsymbol{x})=\lambda \sum\limits_{j=-m}^{m} \alpha^{|j|} \| \boldsymbol{x}-\boldsymbol{G}^{j}\boldsymbol{x} \|_{1},  $$

(17) $$ \mathcal{G}(\boldsymbol{x})=\frac{1}{2}\int_{\mathbb{R}^{p}}|\boldsymbol{x}(t)-\boldsymbol{\bar{x}}(t)|^{2}\mathrm{d}t.  $$

Using the notations above, the problem (7) becomes

(18) $$  \underset{\boldsymbol{x}}{\inf}\, \left\{ \ \mathcal{G}(\boldsymbol{x}) + \mathcal{F}(K\boldsymbol{x}) \right\}.  $$

Now we can apply the Primal-Dual algorithm to minimize the general problem (18), where $\mathcal {F}$ and $\mathcal {G}$ are a convex functions and *K* is a linear operator. Thus, using the saddle point problem [17], we now get the equivalent Primal-Dual problem

(19) $$  \underset{\boldsymbol{x}}{\inf}\,\underset{\boldsymbol{p}}{\sup}\, \left\{ <K\boldsymbol{x},\boldsymbol{p}>+ \mathcal{G}(\boldsymbol{x}) + \mathcal{F}^{*}(\boldsymbol{p}) \right\},  $$

where $\mathcal {F}^{*}$ is the dual of the function $\mathcal {F}$ defined as

(20) $$  \mathcal{F}^{*}(\boldsymbol{p})=\underset{\boldsymbol{p}}{\sup}\, <\boldsymbol{p},\boldsymbol{x}>-\mathcal{F}(\boldsymbol{x}),  $$

where ***p*** is a dual variable such as $\boldsymbol {p} \in \mathbb {R}^{p}$. Then, according to (20) and the definition of using the definition of $\mathcal {F}$ in (16), we can check that

(21) $$  \mathcal{F}^{*}(\boldsymbol{p})=\delta_{\boldsymbol{P}}(\boldsymbol{p})= \left\{ \begin{array}{cc} 0 & \ \boldsymbol{p} \in \boldsymbol{P}\\ +\,\infty & \ \boldsymbol{p} \not\in \boldsymbol{P}, \end{array}\right.  $$

where *P*={***p***:∥***p***∥_*∞*_≤1}, before proceeding to the Primal-Dual algorithm, we have to define the proximity operator functions $\mathcal {F}^{*}$ and *G*. We define firstly the operator $(I+\sigma \partial \mathcal {F}^{*})(\boldsymbol {p})$ using the projection on ***P***, noted *Π*_***P***_, as follows

$$(I+\sigma \partial \mathcal{F}^{*})^{-1}(\boldsymbol{p})= \Pi_{\boldsymbol{P}}(\boldsymbol{p}), $$

where

$$\Pi_{\boldsymbol{P}}(\boldsymbol{p})= \frac{\boldsymbol{p}}{\max(||\boldsymbol{p}||_{\infty},1)}, $$

and

$$||\boldsymbol{p}||_{\infty}=\max_{i,j}|\boldsymbol{p}_{i,j}|. $$

Also we define the operator $(I+\sigma \partial \mathcal {G})^{-1}(\boldsymbol {x})$ using the definition of the function $\mathcal {G}$ as

$$(I+\tau \partial \mathcal{G})^{-1}(\boldsymbol{x})= \frac{\boldsymbol{x}+\varepsilon \boldsymbol{\bar{x}}}{1+\varepsilon}. $$

Now we are ready to implement the Primal-Dual algorithm associated to the problem (7). We summarize this algorithm in the following



Where the operator $K^{\intercal }$ is the adjoint of the operator *K* defined as

$$ K^{\intercal}=\lambda \sum\limits_{j=-m}^{m} \alpha^{|j|} \left(I-\boldsymbol{G}^{-j}\right) $$

### BSS via copula

In this section, we show how to separate instantaneous mixtures after denoising step. The idea is to solve the discrete version of the (1) without noise, defined by

(22) $$  \boldsymbol{x}(n):=A\boldsymbol{s}(n), \;n=1,\ldots,N.  $$

The source signals ***s***(*n*), *n*=1,…,*N*, will be considered as *N* copies of the random source vector ***S***, and then ***x***(*n*), ***y***(*n*):=***Bx***(*n*), *n*=1,…,*N* are, respectively, *N* copies of the random source vector ***X*** and ***Y***:=***BX***.

#### Independent source components

Experimentally, to achieve separation, the idea is to minimize with respect to ***B*** some statistical estimate $\widehat {KL_{m}} \left (\boldsymbol {c}_{\scriptscriptstyle {\prod }}, \boldsymbol {c}_{\boldsymbol {Y}}\right)$ of $KL_{m} \left (\boldsymbol {c}_{\scriptscriptstyle {\prod }}, \boldsymbol {c}_{\boldsymbol {Y}}\right)$, constructed from the data ***y***(1),…,***y***(*N*). Moreover, we can show that the criterion function $\boldsymbol {B}\mapsto KL_{m} \left (c_{\scriptscriptstyle {\prod }}, \boldsymbol {c}_{\boldsymbol {Y}}\right)$ is nonnegative and achieves its minimum value zero iff ***B***=***A***^−1^ (up to scale and permutation indeterminacies), i.e.,

(23) $$  \boldsymbol{A}^{-1} = \arg\underset{\boldsymbol{B}}{\inf}\, KL_{m} \left(\boldsymbol{c}_{\scriptscriptstyle{\prod}}, \boldsymbol{c}_{\boldsymbol{Y}}\right).  $$

The de-mixing matrix is then estimated by

(24) $$  \widehat{\boldsymbol{B}} = \arg\underset{\boldsymbol{B}}{\inf}\, \widehat{KL_{m}} \left(\boldsymbol{c}_{\scriptscriptstyle{\prod}}, \boldsymbol{c}_{\boldsymbol{Y}}\right),  $$

in view of (13), we propose

(25) $$  \widehat{KL_{m}} \left(\boldsymbol{c}_{\scriptscriptstyle{\prod}}, \boldsymbol{c}_{\boldsymbol{Y}}\right):=\frac{1}{N}\underset{n=1}{\overset{N}{\sum}}\log \left(\widehat{c}_{\boldsymbol{Y}}\left(\widehat{F}_{Y_{1}}(y_{1}(n)),\ldots, \widehat{F}_{Y_{p}}(y_{p}(n))\right) \right),  $$

where

(26) $$  \widehat{c}_{\boldsymbol{Y}}(\boldsymbol{u}):=\frac{1}{NH_{1}\cdots H_{p}}\underset{m=1}{\overset{N}{\sum}}\, \underset{j=1}{\overset{p}{\prod}}k\left(\frac{\widehat{F}_{Y_{j}}(y_{j}(m))-u_{j}}{H_{j}} \right),\forall \boldsymbol{u}\in\, [0,1]^{p},  $$

is the kernel estimate of the copula density *c*_***Y***_(.), and $\widehat {F}_{Y_{j}}(x),\, j = 1,\ldots,p$, is the smoothed estimate of the marginal distribution function $F_{Y_{j}}(x)$ of the random variable *Y*_*j*_, at any real value $x \in \mathbb {R}$, defined by

(27) $$  \widehat{F}_{Y_{j}}(x):=\frac{1}{N}\underset{m=1}{\overset{N}{\sum}}K\left(\frac{y_{j}(m)-x}{h_{j}} \right),\, \forall j = 1,\ldots,p  $$

where *K*(.) is the primitive of a kernel *k*(.), a symmetric centered probability density. In our forthcoming simulation study, we will take for the kernel *k*(.) a standard Gaussian density. A more appropriate choice of the kernel *k*(.), for estimating the copula density,can be done according to [20], which copes with the boundary effect. The bandwidth parameters *H*_1_,…,*H*_*p*_ and *h*_1_,…,*h*_*p*_ in (26) will be chosen according to Silverman’s rule of thumb, see [21], i.e., for all *j*=1,…,*p*, we take

(28) $$  \left\{ \begin{array}{ll} H_{j}=\left(\frac{4}{p+2} \right)^{\frac{1}{p+4}}N^{\frac{-1}{p+4}}\widehat{\Sigma}_{j},\\ h_{j}=\left(\frac{4}{3} \right)^{\frac{1}{5}}N^{\frac{-1}{5}}\widehat{\sigma}_{j}, \end{array}\right.  $$

where $\widehat {\Sigma }_{j}$ and $\widehat {\Sigma }_{j}$ are, respectively, the empirical standard deviation of the data $\widehat {F}_{Y_{j}}(y_{j}(1)),\ldots, \widehat {F}_{Y_{j}}(y_{j}(N))$ and *y*_*j*_(1),…,*y*_*j*_(*N*).

The solution $\widehat {\boldsymbol {B}}$ the estimate of the de-mixing matrix, can be computed by a descent gradient algorithm, taking as initial matrix ***B***_0_=*I*_*p*_, the *p*×*p* identity matrix.

We summarize the above methodology in the following algorithm.



#### Dependent source components

The case where the source components are dependent, to achieve separation, the idea is to minimize with respect to ***B*** some statistical estimate $\widehat {KL_{m}} \left (\boldsymbol {c}_{\widehat {\boldsymbol {\theta }}}, \boldsymbol {c}_{\boldsymbol {Y}}\right)$ of $KL_{m} \left (\boldsymbol {c}_{\widehat {\boldsymbol {\theta }}}, \boldsymbol {c}_{\boldsymbol {Y}}\right)$, constructed from the data ***y***(1),…,***y***(*N*), $c_{\widehat {\boldsymbol {\theta }}}(\boldsymbol {u})$ is the copula density modeling the dependency structure of the source components. Obviously, since the source components are assumed to be dependent, $c_{\widehat {\boldsymbol {\theta }}}(\boldsymbol {u})$ is different from the density copula of independence $c_{\scriptscriptstyle {\prod }}(\boldsymbol {u})$. So as before, the separation matrix is estimated by $\widehat {B} = \underset {B}{\arg \min } \widehat {KL_{m}} \left (\boldsymbol {c}_{\widehat {\boldsymbol {\theta }}}, \boldsymbol {c}_{\boldsymbol {Y}}\right)$, leading to the estimated source signals $\widehat {\boldsymbol {y}}(n)=\widehat {\boldsymbol {B}}\,\boldsymbol {x}(n),\quad i=n,\ldots, N$.

We propose to estimate the criterion $\widehat {KL_{m}} \left (\boldsymbol {c}_{\widehat {\boldsymbol {\theta }}}, \boldsymbol {c}_{\boldsymbol {Y}}\right)$ through

(29) $$  \widehat{KL_{m}} \left(\boldsymbol{c}_{\widehat{\boldsymbol{\theta}}}, \boldsymbol{c}_{\boldsymbol{Y}}\right):=\frac{1}{N}\underset{n=1}{\overset{N}{\sum}} \log\left(\frac{\widehat{\boldsymbol{c}}_{\boldsymbol{Y}}\left(\widehat{F}_{Y_{1}}(Y_{1}(n)),\ldots, \widehat{F}_{Y_{p}}(Y_{p}(n)) \right)}{\widehat{\boldsymbol{c}}_{\widehat{\boldsymbol{\theta}}} \left(\widehat{F}_{Y_{1}}(Y_{1}(n)),\ldots, \widehat{F}_{Y_{p}}(Y_{p}(n)) \right)} \right),  $$

The estimates of copula density and the marginal distribution functions are defined as before. The solution $\widehat {\boldsymbol {B}}$ can be computed by a descent gradient algorithm. We obtain then the following algorithm.



## Results and discussion

In this section, both synthetic and real experiment are tested to confirm the performance of our proposed method, and compare it to the BSS via independent component analysis [6] approach. We start firstly by the synthetic experiment.

### Synthetic data

#### Independent source components

**Fig. 1:**
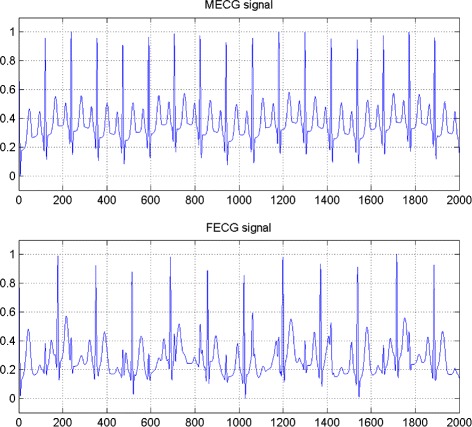
Two synthetic simulated independent MECG and FECG

**Fig. 2:**
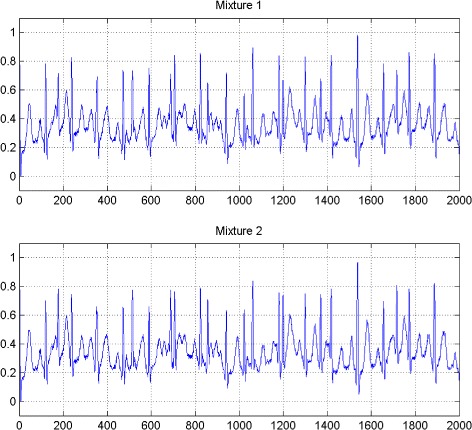
Two noisy mixtures of the FECG and MECG signals

**Fig. 3:**
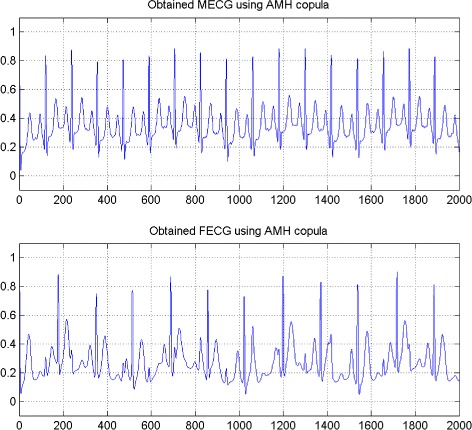
FECG and MECG obtained by the proposed method using independent copula

**Fig. 4:**
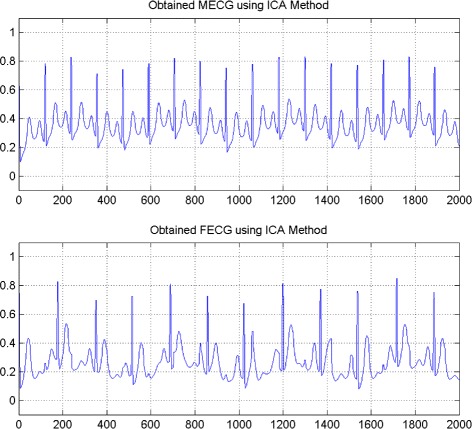
FECG and MECG obtained by ICA method (FECG and MECG are nearly independent)

In the first set of experiments, we use a two synthetic simulated independent MECG and FECG represented in the Fig. 1. We construct two noisy mixtures of the FECG and MECG signals, using a mixing matrix *A*= [1,0.8;0.8,1]. A centered gaussian noise with standard deviation 0.1 was added to the normalized mixtures, so that the signal-to-noise ratio equals –20 dB. The obtained signal mixture are represented in the Fig. 2. In the Fig. 3, we show the separate FECG and MECG obtained by our proposed method, while in the Fig. 4, we present the obtained ones using the BSS via independent component analysis in the separation step and TV approach [22] in the denoising step. For more assessment, the accuracy of source estimation is evaluated through the signal-noise-ratio *SNR*(*dB*) defined by

(30) $$  SNR_{i}:=10\log_{10} \left(\frac{\underset{k=1}{\overset{N}{\sum}} s_{i}(k)^{2}}{\underset{k=1}{\overset{N}{\sum}} \left(y_{i}(k) -s_{i}(k) \right)^{2}}\right),\, i=1,2.  $$

The SNR is a term that refers to the measurement of the level of a signal as compared to the level of noise that is present in that signal.

In other hand, as a measure of the dependence between FECG and MECG signals, we use the Kendall’s *τ*, which is defined in terms of concordance as follows. Let (*Y*_1_,*Y*_2_) and $\left (Y_{1}^{\prime },Y_{2}^{\prime }\right)$ be random vectors, then the population version of Kendall’s *τ* is defined as the difference between the probabilities of concordance and discordance:

(31) $$  \tau=\mathbb{P}\left[ \left(Y_{1}-Y_{1}^{\prime} \right) \left(Y_{2}-Y_{2}^{\prime}\right)>0\right]-\mathbb{P}\left[\left(Y_{1}-Y_{1}^{\prime}\right)\left(Y_{2}-Y_{2}^{\prime}\right)<0\right].  $$

These probabilities can be evaluated by integrating over the distribution of $\left (Y_{2}-Y_{2}^{\prime }\right)$. So that, in terms of copula, Kendall’s tau becomes to

(32) $$  \tau := 4\iint_{[0,1]^{2}}\mathbb{C}(u_{1},u_{2})\mathrm{d} \mathbb{C}(u_{1},u_{2}) -1.  $$

We have *τ*∈[−1,1], and note that, under independence of the margins, we have *τ*=0.

**Fig. 5:**
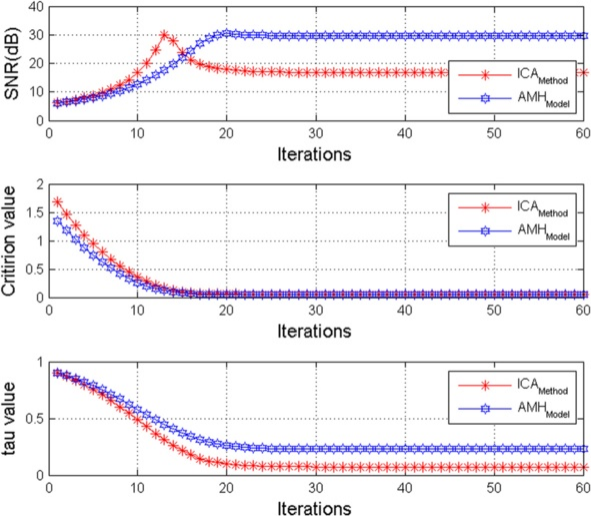
Average output SNRs, criterion value, and Kendall tau, versus iteration number (FECG and MECG sources are nearly independent)

In the Fig. 5 we present the mean of *SNR*’s of the two simulated ECG signals together with the criterion of separation value vs iterations and, in the bottom of the Fig. 5 the associated Kendall’s *τ*. We can see that our criterion and Kendall’s *τ* converges to 0 when the separation is achieved.

#### Dependent source components

**Fig. 6:**
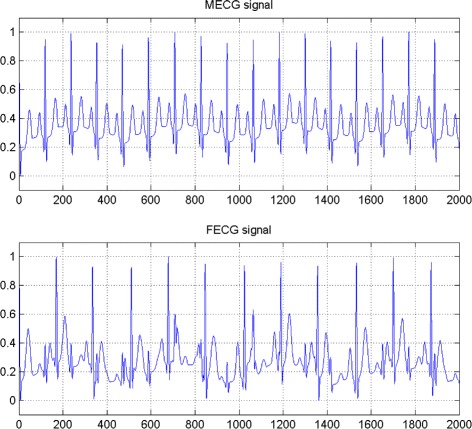
ECG signal vector sources with dependent components generated from AMH copula
- ECG signal vector sources with dependent components generated from Clayton copula with $\widehat {\theta } =0.5$, the estimated Kendall’s *τ* of the source is equal *τ*(***S***)=0.34 (presented in Fig. 7).

In this subsection we show the capability of the proposed method for dependent sources to successfully separate two dependent mixed MECG and FECG signals. We dealt with instantaneous mixtures of two kinds of sample sources:

- ECG signal vector sources with dependent components generated from Ali-Mikhail-Haq (AMH) copula with $\widehat {\theta } =0.4$, the estimated Kendall’s *τ* of the source is equal *τ*(***S***)=0.22 (presented in Fig. 6).

**Fig. 7:**
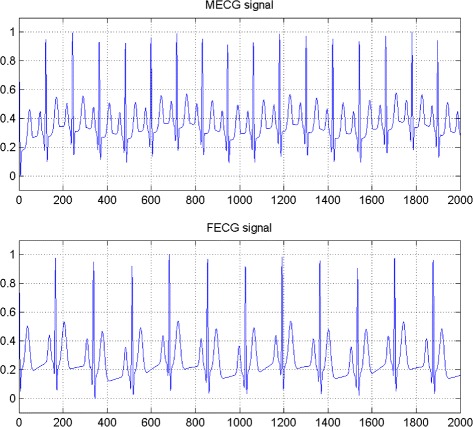
ECG signal vector sources with dependent components generated from Clayton copula

**Fig. 8:**
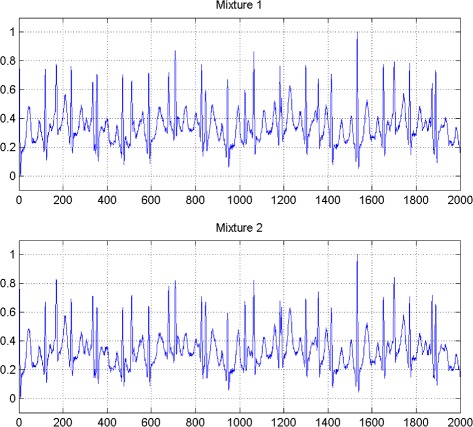
Two noisy mixtures of the dependent FECG and MECG signals using AMH

**Fig. 9:**
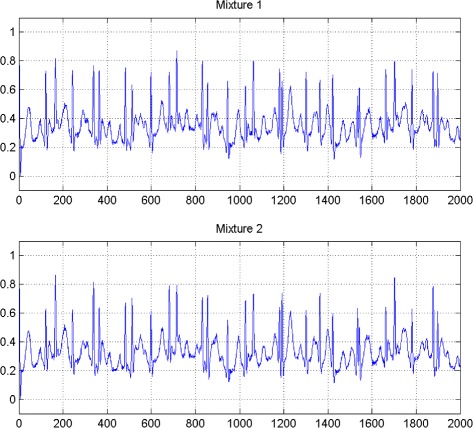
Two noisy mixtures of the dependent FECG and MECG signals using Clayton

**Fig. 10:**
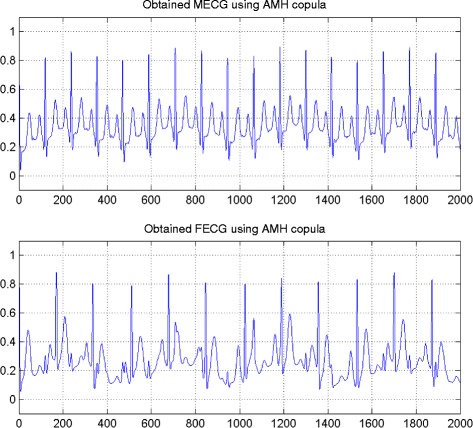
FECG and MECG obtained by the proposed method: AMH copula

**Fig. 11:**
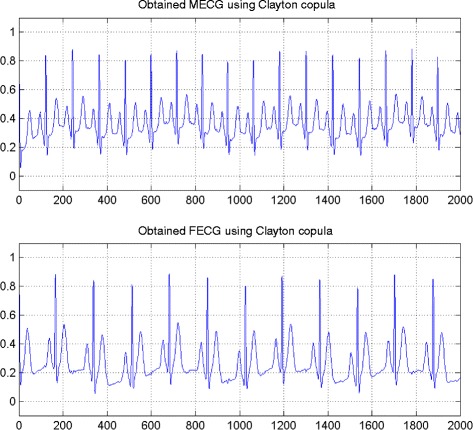
FECG and MECG obtained by the proposed method: Clayton copula

**Fig. 12:**
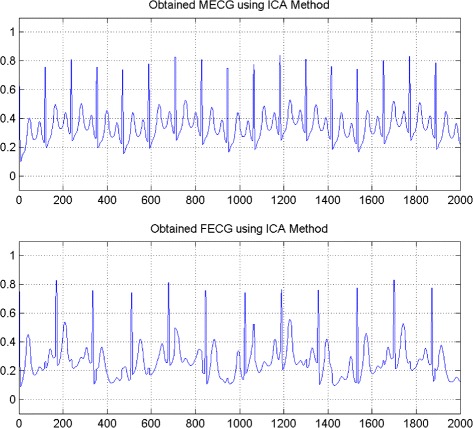
FECG and MECG obtained by ICA method (Dependence is modeled by AMH copula)

**Fig. 13:**
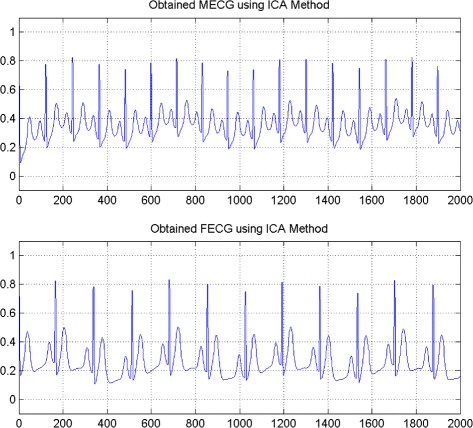
FECG and MECG obtained by ICA method (Dependence is modeled by Clayton copula)

**Fig. 14:**
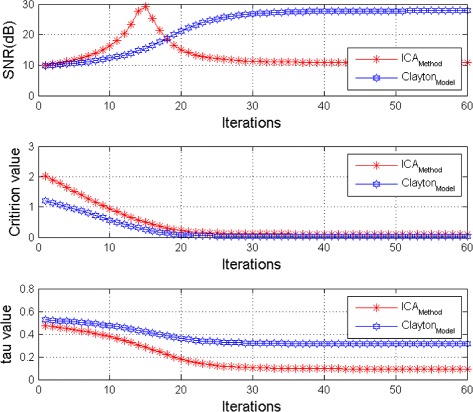
Average output SNRs, criterion value, and Kendall tau, versus iteration number (The dependence of FECG and MECG sources is modeled by AMH copula)

**Fig. 15:**
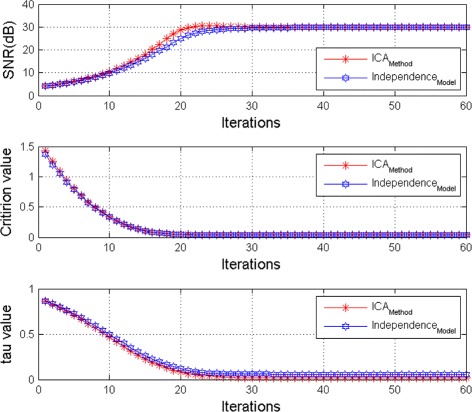
Average output SNRs, criterion value, and Kendall tau, versus iteration number (The dependence of FECG and MECG sources is modeled by Clayton copula)

The Figs. 8 and 9 show the two mixed signals of the MECG, FECG using AMH and Clyton copula respectively with additive Gaussian noise with standard variation *σ*=0.1. In Figs. 10 and 11, we have shown the obtained separate FECG and MECG using AMH and Clyton copula respectively. While in the Figs. 12 and 13 we present the obtained ones using the BSS via independent component analysis associated to AMH and Clyton copula respectively. In the Figs. 14 and 15 we show the mean of *SNR*’s of the two simulated ECG signals associated to AMH and Clyton copula respectively compared with the ICA together with the criterion of separation value vs iterations and, in the bottom of the Fig. 15 the associated Kendall’s *τ*. It can be seen from the simulations that the proposed method is able to separate, with good performance, the mixtures of dependent source components. We can also remark that our criterion converges to 0 when the separation is achieved.

Comparing the proposed method with the ICA for FECG and MECG separation, both methods give a promising results. However in the case of dependence, the ICA fails while our proposed method is still working with a high accuracy.

### Real data

**Fig. 16:**
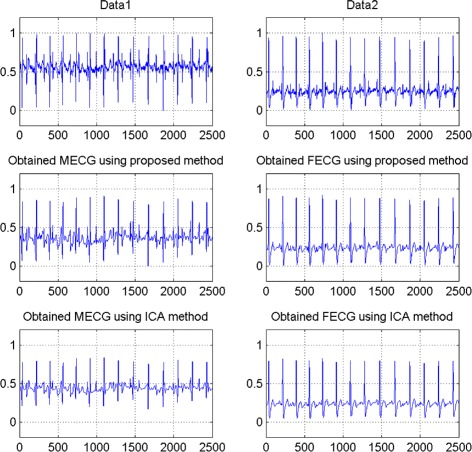
The results of the proposed approach for real ECG signals, compared with the ICA method

The real cutaneous electrode recording used in the experiments is displayed in Fig. 16, which were obtained from the web site^1^ contributed by Lieven De Lathauwer. The signals in Fig. 16 were recorded from eight skin electrodes located on different emplacement of a pregnant woman’s body. The sampling frequency was 250 *Hz* and the sampling time 10 *s*, so each signal is composed of *T*=2500 samples. The first five recordings correspond to electrodes located on the mother’s abdominal region. In this work, we choose only two from the recording five abdominal signals. Firstly, the estimation of the source signals and the mixing matrix via a BSS method. In the middle of the Fig. 16, we show the obtained FECG and MECG signals using our method, while at the bottom of this figure, we present the obtained FECG and MECG using the ICA method. We can see that the proposed approach separate magnificently the two signals without loss of informations compared with the ICA method. Noticed that the proposed method can be applied in other mixed electrophysiological recordings include abnormal signal, electroencephalograms (EEGs) and electrocardiograms (ECGs). This is the objective of a future work.

## Conclusion

A new approach of blind source separation (BSS) for the FECG and MECG separation from two noisy ECG signals was introduced for both independent and dependent ECG sources. The main idea is based on the minimization of the Kullbak-Leibler divergence between copula densities to separate the observed data, and a BTV filter as a pretreatment step for denoising. The accuracy and the consistency of the obtained algorithms are illustrated by simulation, for 2×2 mixture-source.

## Endnote

^1^http://homes.esat.kuleuven.be/~smc/daisy/daisydata.html.

## Abbreviations

SIMPLE: Semi-implicit method for pressure-linked equation
MRI: Magnetic resonance imaging
CFD: Computational fluid dynamics
2D: Two dimensional
